# Complete Genome Sequence of Matlo Bat Lyssavirus

**DOI:** 10.1128/MRA.00241-21

**Published:** 2021-05-20

**Authors:** Colyn S. Grobler, Jessica Coertse, Wanda Markotter

**Affiliations:** a Centre for Viral Zoonoses, Department of Medical Virology, University of Pretoria, Pretoria, South Africa; b Centre for Emerging Zoonotic and Parasitic Diseases, National Institute of Communicable Diseases of the National Health Laboratory Service, Sandringham, South Africa; KU Leuven

## Abstract

The genus *Lyssavirus* includes rabies virus as well as multiple diverse and recently described novel species. Using next-generation sequencing technologies, we have obtained the whole-genome sequence of Matlo bat lyssavirus, which was isolated from a Natal long-fingered bat (*Miniopterus natalensis*) in South Africa.

## ANNOUNCEMENT

Lyssaviruses are bullet-shaped, with an approximately 12-kb-long negative-sense RNA genome encoding five proteins, namely, nucleoprotein (N), phosphoprotein (P), matrix protein (M), glycoprotein (G), and an RNA-dependent polymerase (L). Recently, the lyssavirus species described have significantly increased, with 17 recognized lyssavirus species divided into two distinct phylogroups and several ungrouped viruses (1, 2).

Matlo bat lyssavirus (MBLV) was first detected in the brain of a Natal long-fingered bat (*Miniopterus natalensis*) on 2 September 2015 in Limpopo, South Africa, during targeted bat lyssavirus surveillance (3). Initial analyses of the PCR-amplified nucleoprotein gene sequence (1,353 nucleotides [nt]) indicated that MBLV is most closely related to West Caucasian bat virus (WCBV) (accession number NC_025377), with 80.9% nucleotide identity. Full-genome sequencing was performed using targeted amplicon sequencing on the MiSeq platform (Illumina). The virus was isolated using the standard mouse inoculation test (4). Viral RNA was extracted from mouse brains using TRIzol (Invitrogen); this was followed by first-strand cDNA synthesis using random hexamer primers (Integrated DNA Technologies) and the SuperScript III synthesis system (Thermo Fisher Scientific) (4, 5). The full genome was amplified using four different primer sets (Table 1) and the Phusion high-fidelity DNA polymerase system (New England Biolabs). Amplicons were purified using the Zymoclean gel DNA recovery kit (Zymo Research Corp.) followed by next-generation sequencing (NGS) for each individual purified amplicon using the Nextera XT library preparation kit (Illumina) on a MiSeq sequencing platform (Illumina), with paired-end read lengths of 2 × 300 bp and with 448× coverage obtained. All methods were according to the manufacturers’ instructions unless indicated otherwise. A total of 1,919,813 reads were obtained and assembled using the *de novo* assembly algorithm in CLC Main Workbench v12.0.2 (CLCbio). The entire genome was obtained apart from the 5′ and 3′ termini (71 nt and 50 nt, respectively), which were obtained by RNA circularization, cloning, and sequencing as described previously (5). The assembled viral reads and genomic terminal sequences were mapped to WCBV (GenBank accession number NC025377) as a reference sequence. The consensus sequence obtained was imported into the NCBI open reading frame (ORF) finder, where gene assignment was determined (6). All tools were run with default parameters unless otherwise specified. University of Pretoria Animal Ethics research approval was obtained (approval number EC054-14).

**TABLE 1:** Primers designed for use during MiSeq amplicon sequencing of MBLV

Primer set and name	Primer binding position^*a*^	Sequence (5′ to 3′)
Set 1
JW12 (7)	55–74	ATG TAA CAC CYC TAC AAT G
CG LYSSA S1 R	4031–4049	TRA ACA DBC CTC TYT CAT C
Set 2
CG LYSSA S2 F	2771–2793	TCT GGB AAY MGA MGR ATG ATA GG
CG LYSSA S2 R	6941–6967	AYT TTT TCA TAT GGA CTT GAT CRT AMA
Set 3
CG LYSSA S3 F	6347–6364	TRG AYT GGG ATG ARG ARA
CG LYSSA S3 R	9569–9587	CTV ACW GAG ATA TGA GAC A
Set 4
CG LYSSA S4 F	8671–8690	GAG GAY CCW ACC ACH CTS AA
001lys (8)	1–15	ACG CTT AAC GAM AAA

a Primer binding positions are relative to the WCBV sequence (GenBank accession number NC025377) except for 001lys and JW12, for which the binding positions are relative to the Pasteur virus sequence (M13215).

**FIG 1:**
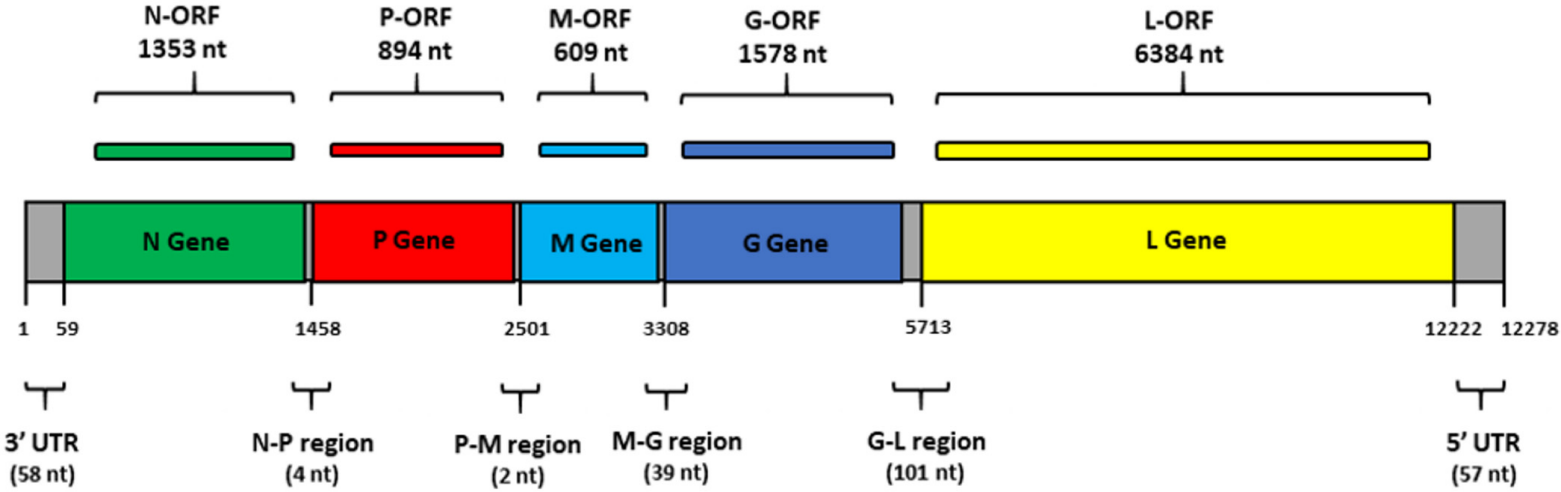
Genome characteristics determined for MBLV. The N, P, M, G, and L protein gene lengths are shown. IGS and 5′ and 3′ untranslated region (UTR) lengths are indicated in parentheses.

The genomic organization of MBLV was found to be similar to that of known lyssaviruses, with a complete genome length of 12,278 nt (GC content, 41.85%) (Fig. 1). MBLV was found to be most similar to WCBV, with a complete genome similarity of 78.9%. Gene positions and lengths were identified with the NCBI ORFfinder and subsequently compared to known lyssavirus sequences. Gene lengths and intergenic spacers (IGSs) were found to be similar to those of the unclassified lyssaviruses, with the G-L IGS (101 nt) being significantly larger than those of phylogroup I and II lyssaviruses. The level of similarity of known antigenic regions between MBLV and the other unclassified lyssaviruses also provided initial evidence that cross neutralization will most probably not occur when immunized hosts are exposed to this virus.

### Data availability.

The complete genome sequence of MBLV has been deposited in GenBank under accession number MW653808. Raw NGS sequence reads have been deposited in the SRA under accession number PRJNA708137.
